# Incidence and risk factors of osteoporotic status in outpatients who underwent gastrectomy for gastric cancer

**DOI:** 10.1002/jgh3.12347

**Published:** 2020-04-23

**Authors:** Tsutomu Namikawa, Keiichiro Yokota, Jun Iwabu, Masaya Munekage, Sunao Uemura, Shigehiro Tsujii, Hiromichi Maeda, Hiroyuki Kitagawa, Takashi Karashima, Masamitsu Kumon, Keiji Inoue, Michiya Kobayashi, Kazuhiro Hanazaki

**Affiliations:** ^1^ Department of Surgery Kochi Medical School Nankoku Japan; ^2^ Department of Urology Kochi Medical School Nankoku Japan; ^3^ Department of Surgery Noichi Central Hospital Kohnan Japan; ^4^ Department of Human Health and Medical Sciences Kochi Medical School Nankoku Japan

**Keywords:** bone disorder, dual X‐ray mineral absorptiometry, gastric cancer, osteoporosis

## Abstract

**Background and Aim:**

Disorders in bone metabolism have long been recognized as typical sequelae of gastrectomy; however, the pathogenesis has not been fully elucidated, resulting in a variation of reported incidence. This study aimed to evaluate current bone health by measuring bone mineral density (BMD) in patients treated by gastrectomy for gastric cancer, with a focus on incidence and risk factors of osteoporosis.

**Methods:**

The study enrolled 81 patients who underwent gastrectomy for gastric cancer at Kochi Medical School. BMD of the lumbar spine was measured by dual‐energy X‐ray mineral absorptiometry, with the results expressed as a percentage of the young adult mean (YAM). Clinical data were also obtained to investigate associations with BMD.

**Results:**

Of the 81 study patients, 12 (14.8%) were deemed to have osteoporosis, defined by a percentage of YAM <70, with a dominance of females over males (66.7% *vs* 17.4%; *P* < 0.001). The median body weight, hemoglobin concentration, and serum alkaline phosphatase (ALP) level of the patients with osteoporosis was significantly lower than in those with a percentage of YAM ≥70 group (39.6 kg *vs* 53.1 kg, *P* < 0.001; 10.9 mg/dL *vs* 12.5 mg/dL, *P* = 0.010; 210 U/L *vs* 251 U/L, *P* = 0.002). Further analyses revealed a significant positive correlation between body weight and percentage of YAM (r = 0.441, *P* < 0.001). Despite the administration of bisphosphonates in these patients during this study, one acquired a bone fracture.

**Conclusion:**

Osteoporosis was found in 14.8% of postoperative gastric cancer patients, with female gender, low body weight, and low ALP proposed as risk factors for osteoporosis and thus future bone fracture.

## Graphical abstract

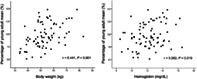

We found osteoporosis in 14.8% of outpatients receiving periodic follow‐up physical examinations after gastrectomy for gastric cancer, and identified low BMD as a clear indicator of increased bone fragility and risk for fracture in these patients. In these patients, female gender, low body weight, low hemoglobin, and low ALP were potential risk factors for osteoporosis.

## Introduction

Gastric cancer is one of the most common malignant tumors and the seventh leading cause of cancer‐related death worldwide and the second most frequent cause of cancer‐related deaths in Japan.^1^, ^2^, ^3^ Gastrectomy with regional lymphadenectomy is a common treatment component for gastric cancer; however, it is also a known risk factor for osteoporosis, a skeletal disorder characterized by decreased bone mass with accompanying microarchitectural damage.^4^, ^5^

Osteoporosis can also lead to impaired skeletal strength and elevated susceptibility to fractures because it is a progressive skeletal disease manifested by decreased bone mineral density (BMD) accompanying bone fragility and collateral damage to the bone microarchitecture.^4^, ^6^ Actually, a recent study indicated a high cumulative incidence of fractures in gastric cancer patients who have undergone gastrectomy, with a consequent decrease in quality of life and increase in economic burden, both for the individual patients and society.^7^ Although disorders in bone metabolism have long been recognized as typical sequelae of gastrectomy, the pathogenesis has not been fully elucidated, resulting in a variation of reported incidence.

BMD has been widely accepted as a surrogate parameter for the diagnosis of osteopenia and osteoporosis.^5^, ^6^ Numerous clinically accepted methods exist to determine BMD and thus confirm a suspected osteoporotic status, with single X‐ray absorptiometry being the commonly used radiographic bone mass measurement technique. Dual‐energy X‐ray mineral absorptiometry (DEXA), considered the gold standard, includes some advantage such as a shortened examination time, higher accuracy, precision of technique, and removal of errors due to source decay correction.^8^ Nevertheless, assessing the status of a metabolic bone disorder using DEXA for outpatients who underwent gastrectomy for gastric cancer remains controversial. Therefore, we retrospectively analyzed current bone health, evaluating the incidence and risk factors of osteoporosis based on BMD measurements using DEXA in outpatients who underwent gastrectomy for gastric cancer.

## Methods

### *Patients*

Eighty‐one patients who underwent curative gastrectomy for gastric cancer at Kochi Medical School and had survived without disease recurrence were enrolled in this study. The subjects came to the outpatient department of surgery to receive periodic follow‐up physical examinations after gastrectomy during the period of January to December 2018. We rated metabolic bone disorder by the extent of bone density reduction.

In accordance with the guidelines of the Japanese Gastric Cancer Association,^9^, ^10^ D2 lymph node dissection was performed in patients with advanced stage disease. Following distal gastrectomy, Billroth I and Roux‐en‐Y reconstruction was performed in 45 and 6 patients, respectively. Roux‐en‐Y reconstruction was performed in all patients undergoing total gastrectomy. All patients underwent endoscopy, abdominal computed tomography, chest X‐ray, and blood chemistry testing during the last month of follow‐up to detect possible malignancy recurrence. The exclusion criteria were presence of other cancer types; another medical condition known to affect BMD including concurrent or previous treatment with bisphosphonates; or any other bone‐active drugs, chronic kidney disease, and chronic liver disease.

Biochemical measurements from peripheral blood samples were collected and analyzed for serum concentrations of hemoglobin, white blood cell and platelet counts, and alkaline phosphatase (ALP).

This study was approved by the Institutional Review Board of Kochi Medical School, Kochi, Japan, and was conducted according to the principles of the Declaration of Helsinki. Informed consent was obtained from all patients.

### *BMD* *analysis*

The bone mineral content and BMD of the lumbar spine (L2‐4) was measured by DEXA using a Hologic QDR4500SL bone densitometer (Hologic, Waltham, MA, USA), which provides simultaneous measurement of X‐rays with two different energies through the body. DEXA images were automatically analyzed, and reports were generated using vendor‐specific software, with the results expressed as the percentage of young adult mean (YAM).

Measurements were classified into two groups: percentage of YAM <70 and percentage of YAM ≥70 group, based on the Japanese diagnostic criteria of primary osteoporosis,^11^ with −2.5 SD in the T‐score of the World Health Organization (WHO).^12^ T‐score is the number of SDs between the patient's mean BMD and the mean of the population compared with reference populations matched by gender and race.^12^

### *Statistical analysis*

We tested differences between mean values for the two groups of patients for significance using the Mann–Whitney *U* test for continuous variables and Pearson's Chi‐square test for categorical variables. Statistical analyses were performed using SPSS for Windows, version 22.0.

## Results

### *Patients characteristics*

Table 1 summarizes the clinical characteristics of the study patients who underwent gastrectomy for gastric cancer. The cohort comprised 61 men and 20 women with a median age of 72 years (range 31–92 years). Surgical treatment comprised total gastrectomy in 30 and distal gastrectomy in 51 patients. Twenty‐one patients were diagnosed with stage I gastric cancers, with 22, 36, and 2 patients found to have stages II, III, and IV disease, respectively. Fifty‐seven patients received adjuvant chemotherapy following complete resection of a gastric cancer using an S‐1‐based regimen according to the results of pivotal randomized phase III trials.^13^, ^14^ All patients had an Eastern Cooperative Oncology Group Performance Status (ECOG‐PS) of 0.

**Table 1:** Clinical patient characteristics

Age, years, median (range)	72 (31–92)
Gender	
Male	61
Female	20
Operation	
Distal gastrectomy	51
Total gastrectomy	30
Disease stage	
I	22
II	21
III	36
IV	2
Postoperative date, months	13.8 (0–36.2)
Body weight, kg	51.9 (33.7–83.0)
Body mass index	20.1 (14.4–33.9)
Hemoglobin (mg/dL)	12.3 (8.5–16.5)
White blood cell count (/mm^3^)	5250 (2700–12 300)
Platelet (×104/mm^3^)	19.5 (9.5–41.9)
Alkaline phosphatase (U/L)	238 (106–1277)

The median postoperative time, body weight, and body mass index was 13.8 months (range, 0–36.2 months), 51.9 (range 33.7–83.0 kg), and 20.1 (14.4–33.9), respectively. The median hemoglobin level, white blood cell count, platelet count, and alkaline phosphatase (ALP) concentration across all 81 patients was 12.3 mg/dL (range, 8.5–16.5), 5250/mm^3^ (range, 2700–12 300), 19.5 × 10^4^/mm^3^ (range, 9.5 × 10^4^–41.9 × 10^4^), and 238 U/L (range, 106–1277), respectively.

### *Clinical characteristics depending on young adult mean*

Based on the results of BMD measurements using DEXA, patients were divided into two groups: percentages of YAM <70 and ≥70. There were 12 patients with a percentage of YAM <70; thus, the incidence of osteoporosis was 14.8% across the 81 patients, and 1 of the 12 showed a bone fracture in the distal side of the right radius caused by accidental slipping and falling down postgastrectomy. All the 12 patients diagnosed with osteoporosis were administered a bisphosphonate formulation.

Table 2 describes the patients' clinical characteristics depending on percentage of YAM. The osteoporosis group was female dominant (66.7% *vs* 17.4%; *P* < 0.001) and had a significantly lower median body weight than the patients with the percentage of YAM ≥70 (39.6 kg *vs* 53.1 kg; *P* < 0.001). The median hemoglobin and ALP levels of the patients with the percentage of YAM <70 group were also significantly lower than those not diagnosed with osteoporosis (10.9 mg/dL *vs* 12.5 mg/dL, *P* = 0.010 and 210 U/L *vs* 251 U/L, *P* = 0.002, respectively). There were no significant differences between the groups with regard to age, surgical method, disease stage, postoperative period, body mass index, white blood cell count, and platelet count.

**Table 2:** Clinical patient characteristics depending on young adult mean (YAM)

	Percentage of YAM <70 (*n* = 12)	Percentage of YAM ≥70 (*n* = 69)	*P* value
Age, years, median (range)	73 (49–88)	72 (31–92)	0.364
Gender			<0.001
Male	4	57	
Female	8	12	
Operation			0.719
Distal gastrectomy	7	44	
Total gastrectomy	5	25	
Disease stage			0.957
I	2	16	
II	3	18	
III	6	30	
IV	1	5	
Postoperative date, months	13.8 (0–36.2)	16.1 (0–103.5)	0.235
Body weight, kg	39.6 (33.7–58.0)	53.1 (34.4–83.0)	<0.001
Body mass index	19.3	20.3	0.392
Hemoglobin (mg/dL)	10.9	12.5	0.010
White blood cell count (/mm^3^)	5350 (2700–9400)	5300 (2700–12 300)	0.968
Platelet (×10^4^/mm^3^)	17.9 (10.5–25.6)	19.6 (9.5–41.9)	0.617
Alkaline phosphatase (U/L)	210 (122–347)	251 (106–1277)	0.002

### *Relationship between patient young adult mean and patient characteristics*

**Figure 1:**
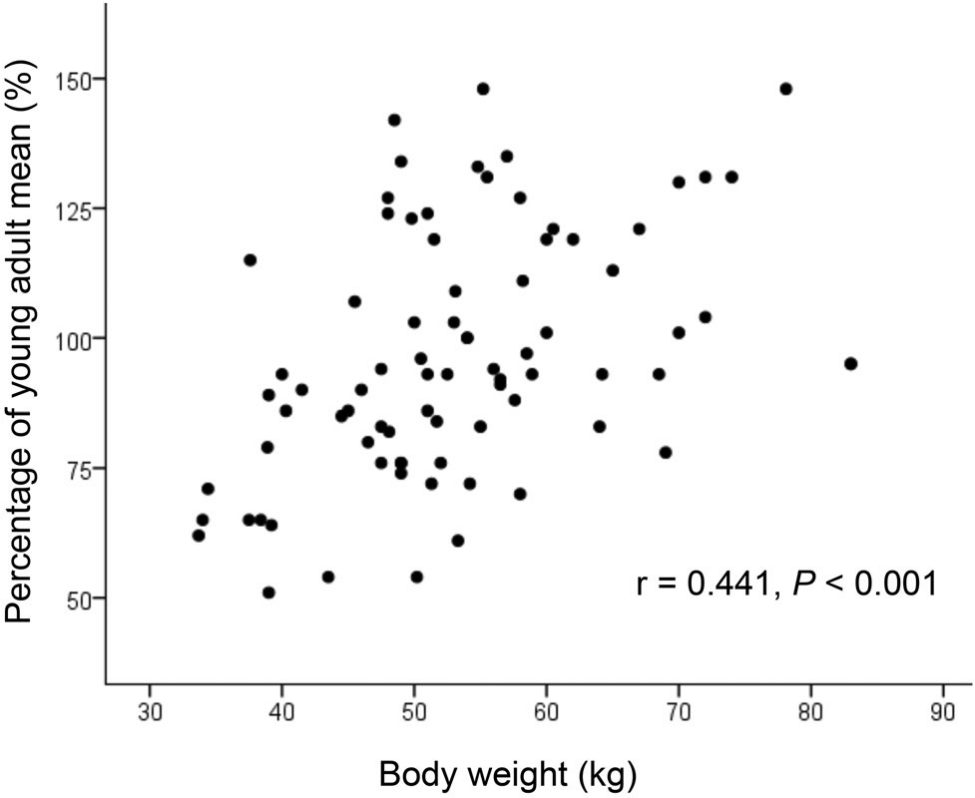
Scatter plot of patient percentage of young adult mean (YAM) and body weight. Percentage of YAM showed a significant positive correlation with body weight (r = 0.441, *P* < 0.001).

**Figure 2:**
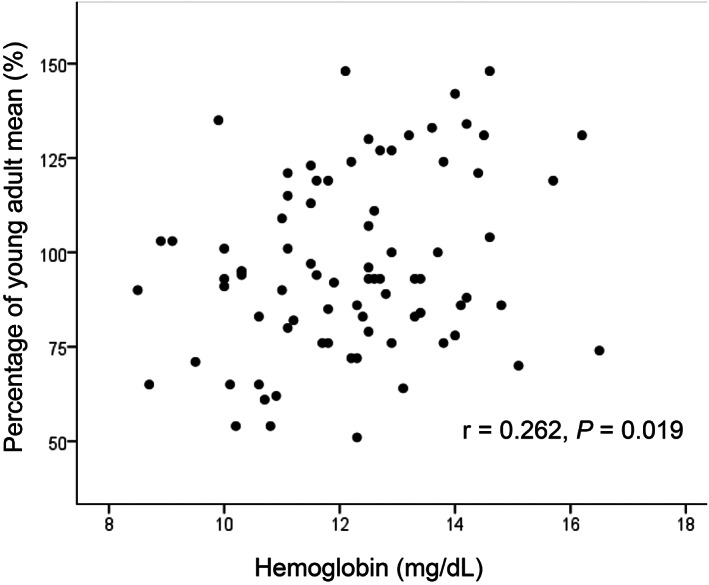
Scatter plot of patient percentage of young adult mean (YAM) and hemoglobin level. There was a slight, but significant, positive correlation between percentage of YAM and hemoglobin level (r = 0.262, *P* = 0.019).

**Figure 3:**
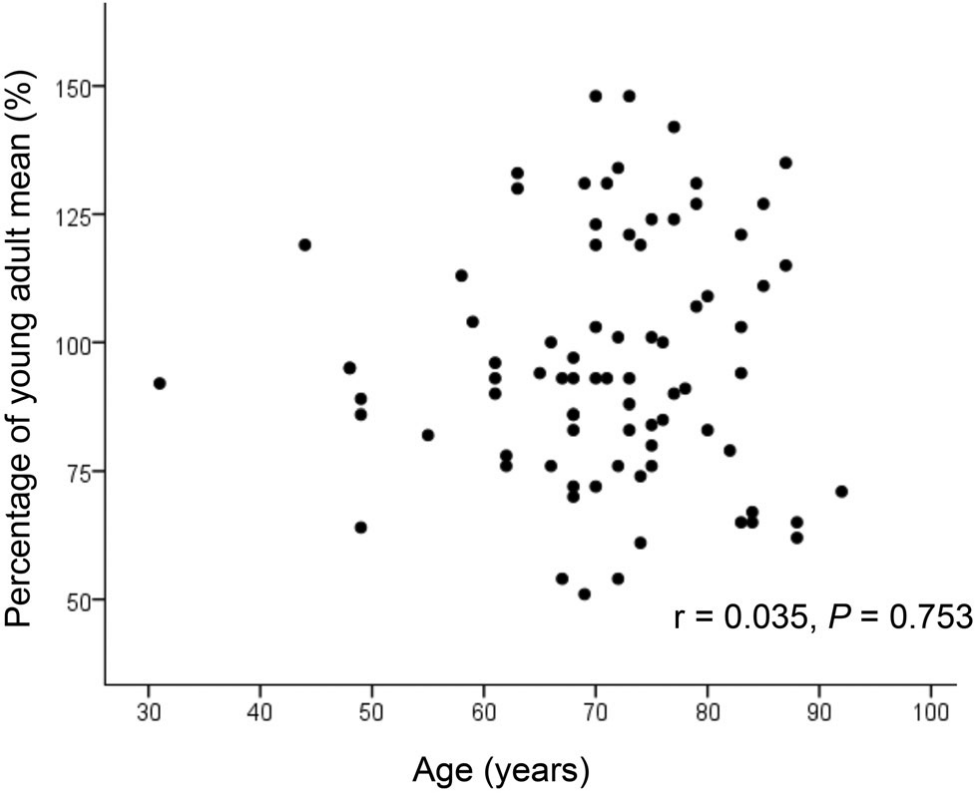
Scatter plot of patient percentage of young adult mean (YAM) and age. There were no significant correlations between percentage of YAM and age (r = 0.035, *P* = 0.753).

**Figure 4:**
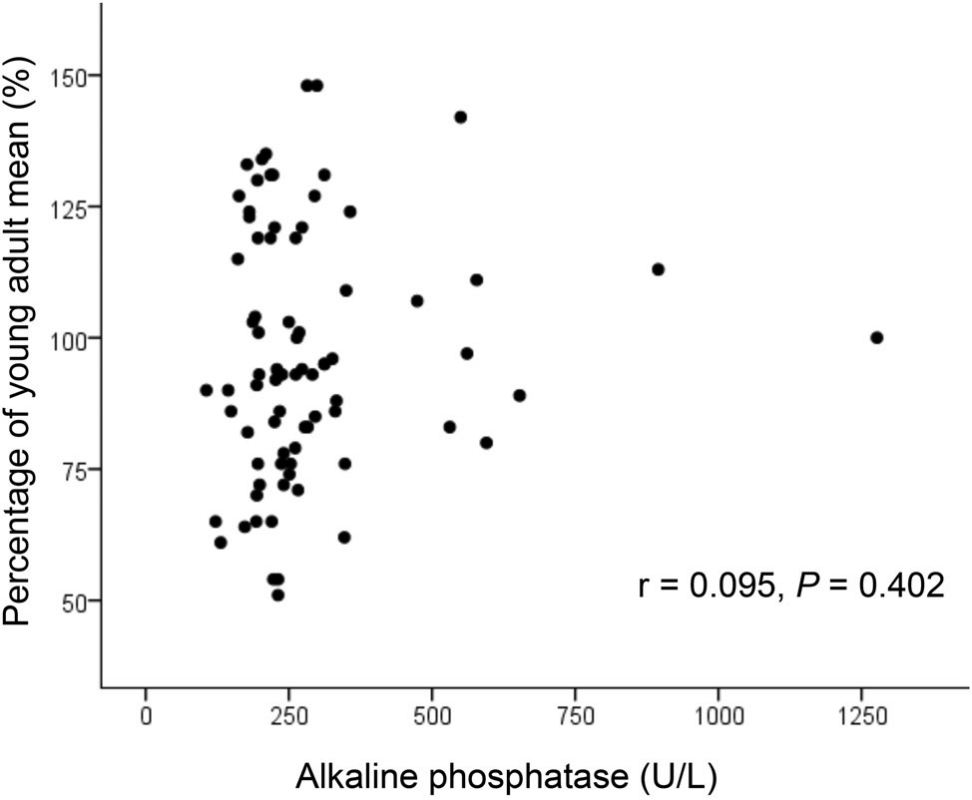
Scatter plot of patient percentage of young adult mean (YAM) and alkaline phosphatase (ALP). There were no significant correlations between percentage of YAM and ALP (r = 0.095, *P* = 0.402).

**Figure 5:**
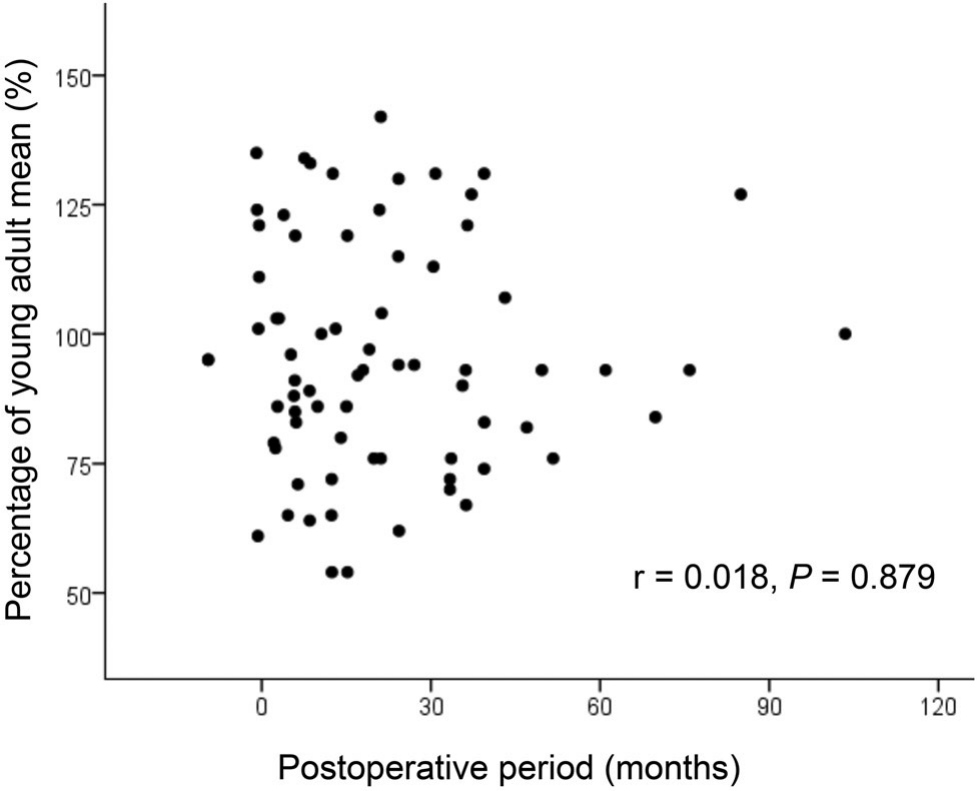
Scatter plot of patient percentage of young adult mean (YAM) and postoperative period. There was no significant correlation between percentage of YAM and postoperative period (r = 0.018, *P* = 0.879).

Figures 1, 2, 3, 4, 5 represent the correlations between BMD of the lumbar vertebra and percentage of YAM, showing a significant positive correlation with body weight (r = 0.441, *P* < 0.001; Fig. 1) and hemoglobin level (r = 0.262, *P* = 0.019; Fig. 2). There were no significant correlations between percentage of YAM and other factors, including age, ALP, and postoperative period (Figs 3, 4, 5).

## Discussion

We found osteoporosis in 14.8% of outpatients receiving periodic follow‐up physical examinations after gastrectomy for gastric cancer and identified low BMD as a clear indicator of increased bone fragility and risk of fracture in these patients. In these patients, female gender, low body weight, low hemoglobin, and low ALP were potential risk factors for osteoporosis. This study indicates the importance of adequate follow‐up examination and BMD monitoring of patients after gastrectomy to ensure accurate and early detection of osteopenia and osteoporosis.

Postoperative follow up of patients after gastric cancer resection is organized by oncologists and surgeons to rule out recurrence; however, while the combination of radical surgery and adjuvant therapy has improved survival rates, the incidence and risk factors for bone disease after gastric cancer resection have received little research attention.^15^ The current study highlights the importance of analyzing bone health in gastric cancer patients because bone loss and the risk of osteoporosis and fractures can have a significant negative impact on the lives of long‐term gastric cancer survivors.^16^

DEXA is a widely accepted radiological tool used to detect early osteoporotic changes in bones, and it is widely used to diagnose osteoporosis with a higher accuracy than plain radiographs of the skeletal system.^17^ A previous study investigating the effect of total gastrectomy on BMD and bone mineral metabolism could not detect osteopenic patients based on laboratory tests alone without measuring BMD.^18^ Other previous reports also suggest that peripheral blood tests such as those for serum calcium or 25‐hydroxyvitamin D levels are of no value in diagnosing reduced bone mass.^6^ It is also currently recognized that all patients being considered for treatment of osteoporosis should be counseled on risk factor reduction, including the importance of calcium, vitamin D, and exercise, as part of any treatment program for osteoporosis.^19^ Indeed, the National Osteoporosis Foundation (NOF) recommends that pharmacologic treatment including bisphosphonates, estrogens and/or hormone therapy, parathyroid hormone, and receptor activator of nuclear factor kappa‐B (RANK) ligand inhibitor should be considered in patients with osteoporosis who have a high fracture risk.^19^ In the present study, we started the administration of bisphosphonates in 12 patients who were diagnosed with osteoporosis during this study, and 1 of them had a bone fracture, although the median postoperative follow up in the present study was relatively short at 13.8 months. Recently, Atsumi *et al*.^20^ prospectively evaluated changes in bone metabolism after gastrectomy for gastric cancer and demonstrated a significant decrease in BMD as early as 1 year after gastrectomy and for up to 2 years.^20^ We thus propose the need for more detailed postoperative surveillance and pharmacological intervention over the long term to prevent bone fracture in gastric cancer patients.

Because there were no data on BMD before surgery in the present study, when BMD begins to change and the extent of such change remain unknown. A recent study prospectively investigated the changes in bone metabolism after gastric cancer surgery compared with preoperative BMD, demonstrating a significant decrease in BMD for up to 24 months after gastrectomy, not only for 12 months.^20^ Although the previous studies demonstrated that pharmacologic treatment is effective for the bone disorders after gastric cancer surgery, the administration period remains to be established.^20^, ^21^

Previous studies showed that many factors, including age, gender, race, genetics, reproductive status, low calcium intake, and exercise, affect BMD.^22^, ^23^ In addition, osteoporosis is a recognized complication of specific diseases and disorders such as chronic liver disease, low femoral neck BMD, and chronic obstructive pulmonary disease.^23^ The results of our study identified female gender, low body weight, and low serum ALP as risk factors for osteoporosis in patients with gastric cancer who had undergone gastrectomy. Jeong et al. recently reported the relative risk for osteoporosis in gastric cancer survivors compared to the general population free of cancer, demonstrating results almost the same as ours.^24^ Yoo et al. identified risk factors associated with osteoporosis in long‐term gastric cancer survivors, which were older age, higher alkaline phosphatase levels before gastrectomy, and marked weight loss after gastrectomy.^25^ Furthermore, Imamura et al. demonstrated that the BMD loss in the patients with Roux‐en‐Y reconstruction was significantly greater than that those with Billroth‐I reconstruction during the first 3 years after surgery.^26^ Therefore, the reconstruction method after gastrectomy might be a cautionary factor for postoperative bone metabolism.

In the present study, patients with and without osteoporosis showed a significant difference in body weight, hemoglobin level, and serum ALP, supporting a previous report that low body weight correlates with increased risk of fracture, especially of the hip.^27^ Positive correlation of body weight with BMD might therefore contribute to multidimensional reasons for such a correlation, including mechanical load reducing bone resorption and stimulating bone formation, hormones increasing osteoprotegerin expression, and nutritional status affecting bone remodeling.^28^ Several studies involving large sample cohorts of older community‐dwelling persons also showed that low hemoglobin levels are negatively associated with BMD, especially in females,^29^, ^30^ supporting our present finding in postgastrectomy patients. Although no clear mechanism of such an osteoporotic tendency has been established, increased oxidative stress and extracellular acidification under hypoxic conditions are estimated to influence bone formation and remodeling.^29^

ALP includes various isoenzymes from other tissues such as bone and liver; thus, an accurate diagnosis of osteoporosis or bone weakness is usually not possible on the basis of ALP alone.^31^ To this end, Mukaiyama *et al*.^31^ examined serum ALP in a group of 626 postmenopausal osteoporotic women before and after treatment with a bisphosphonate, demonstrating that elevated ALP in postmenopausal women is mainly caused by high bone turnover.^31^ Thus, although ALP is not a suitably specific parameter for monitoring changes in bone formation, it might provide a good indication of the extent of new bone formation and osteoblast activity.^32^ In addition, a recent study demonstrated a significant association between ALP level and patient survival following chemotherapy for gastric cancer,^33^ while several retrospective studies of bone disorders occurring after gastric cancer surgery demonstrated that bone loss was generally greater in those patients receiving chemotherapy.^6^

There were notable limitations in the present study. First, selection bias may have influenced survival data due to the retrospective nature of this study. Second, this study was conducted in a single institution with a relatively small number of subjects, thus possibly introducing patient selection bias. Third, we had no data of BMD using DEXA before gastrectomy due to the cross‐sectional study that did not include longitudinal data. Therefore, the results of this study should be interpreted cautiously. Further studies with adequate statistical power and a larger number of patient subgroups are therefore needed to determine the amount of bone loss occurring in the postgastrectomy patients.

In conclusion, the incidence of osteoporosis is high in patients with gastric cancer who had undergone gastrectomy, with female gender, low body weight, and low serum ALP identified as risk factors for osteoporosis that could increase the risk of bone fracture. Further studies are needed to confirm and update our results such that more intensive surveillance pharmacologic interventions can be introduced for gastric cancer patients.

## Declaration of conflict of interest

None of the authors received funding or have any competing interests to disclose.
